# Protective effect of rifampicin and clindamycin impregnated devices against *Staphylococcus *spp. infection after cerebrospinal fluid diversion procedures

**DOI:** 10.1186/1471-2377-10-93

**Published:** 2010-10-12

**Authors:** Raquel Gutiérrez-González, Gregorio R Boto, Cristina Fernández-Pérez, Náyade del Prado

**Affiliations:** 1 Department of Neurosurgery, Hospital Universitario Clínico San Carlos. Prof. Matin Lagos s/n, 28040 Madrid, Spain; 2 Department of Preventive Medicine, Hospital Universitario Clínico San Carlos. Prof. Matin Lagos s/n, 28040 Madrid, Spain

## Abstract

**Background:**

Infection is a major complication of cerebrospinal fluid shunting procedures. The present report assesses the efficacy of such catheters in both shunts and external ventricular drains (EVDs) against infection and particularly against *Staphylococcus *spp. infection.

**Methods:**

All shunt and EVD procedures performed by means of antibiotic-impregnated catheters (AICs) and non-AICs during the period of study were registered. In cases of shunt procedures, a minimal follow-up of 90 days was considered, as well as de novo insertion and catheter revisions. Single valve revisions were not included. In cases of EVD procedures, those catheters removed before the fifth post-insertion day were not included. A total of 119 cerebrospinal fluid shunting procedures performed with AICs were studied in comparison with 112 procedures performed by means of non-AICs.

**Results:**

Antibiotic-impregnated catheters were associated with a significant decrease in both overall and staphylococcal infection (*p *= 0.030 and *p *= 0.045, respectively). The number needed to treat for AICs was 8 to prevent one infection and 14 to prevent one staphylococcal infection. When comparing with shunts, the use of EVDs was associated with a 37-fold increased likelihood of infection.

**Conclusions:**

Antibiotic-impregnated catheters are a safe and helpful tool to reduce CSF shunting device-related infections.

## Background

Infection is a major complication of cerebrospinal fluid (CSF) shunting devices. It involves higher morbidity and mortality for patients but higher costs for the hospital as well [1-10]. In the last decades preventive measures have focused on surgical technique, the use of peri-operative antibiotic prophylaxis and implanted devices. Catheters coverage with antibiotics or antiseptics has been one of the latest measures to be considered [11-35]. The aim of such measure is the slow delivery of the drug along the early postoperative period (which is theoretically the moment of higher risk) in order to avoid bacterial adherence to the catheter [18,36,37]. One of the first studies in this regard was published by Gower et al. [38] in 1985. The author employed catheters soaked up in Bacitracin A for 30 minutes, which resulted in a reduction of 54% of *Staphylococcus epidermidis *adherence to the catheter surface.

The development of CSF shunting devices impregnated with antiseptic or antibiotic agents has focused on preventing *Staphylococcus *spp. infections and particularly those ones caused by biofilm-producer *Staphylococci *strains, since these latter have been shown to be the main causative pathogens [39]. Although many authors defend the protective effect of AICs against infection in shunt procedures, there is short experience with external ventricular drains (EVDs). Besides that, only overall infection effect has been considered in most of the reports published to date and concerning the protective effect of AICs [11-15,18,19,25-27,29].

Clinical observation at our centre has led to believe that AICs are not only associated with a lower risk of staphylococcal infection, but to a higher risk of gram-negative bacilli (GNB) infection. This suspicion was partially confirmed in a preliminary study carried out at our centre [17]. The aim of the present study is to assess the efficacy of rifampicin and clindamycin impregnated CSF shunting devices against infection and particularly staphylococcal infection, as well as the effect of such catheters against GNB infection.

## Methods

A single-centre, retrospective cohort study was designed to assess the efficacy of EVDs and shunts impregnated with rifampicin and clindamycin against infection (Bactiseal; Codman, Johnson & Johnson, Raynham, MA, USA) when comparing them with non-impregnated devices.

### Patients selection

All patients who underwent CSF shunt placement at our centre during the period of study from January 1^st ^2004 to October 31^st ^2008 were reported. The procedures included both de novo insertion and shunt revisions (ventricular catheter, distal catheter or full revision). Single valve substitution was not included, as this part of the shunt is never impregnated and so, individual substitution should not influence in the event to be studied. A minimal follow-up of 90 days was considered. All patients who underwent an EVD insertion at our centre and kept the catheter for at least 5 days during the period of study from January 1^st ^2006 to October 31^st ^2008 were also included. Emergency and elective procedures were considered. Both dates of initial data collection correspond to the introduction of AICs in the regular clinical practice at our centre. Prior approval for AICs use was obtained from the institutional review board. Procedures were assigned to the treatment cohort when rifampicin (0.054%) and clindamycin (0.15%) impregnated catheters ("Bactiseal"; Codman, Johnson & Johnson, Raynham, MA, USA) were employed and to the control cohort when non-AICs were implanted.

The presence of known allergy to rifampicin or clindamycin was the only strict exclusion criterion for the insertion of AICs. Clinical indication for shunt procedures was CSF shunting in every case. In EVDs, need of intracranial pressure monitoring was also included. The choice of AICs versus non-AICs was a decision solely made by the neurosurgeon in charge in all cases. No changes were made in surgical technique or peri-operative patient care. Written informed consent was obtained from patients or representatives in every elective procedure and when possible in emergency procedures.

A total of 231 procedures (119 shunts and 112 EVDs) performed in 171 patients were included for final analysis. Antibiotic-impregnated catheters were employed in 119 of them, using non-AICs in the 112 remaining procedures.

### Dependent variable: infection

Infection was defined by positive result of CSF or/and surgical wound exudation culture, independently from catheter culture result. Positive result required the same microorganism grew on two consecutive positive samples when contamination was suspected.

### Independent variables

Epidemiological, clinical and surgical variables were registered from every procedure included in the database (*Table *1).

**Table 1:** Independent variables

Shunts	EVDs
*EPIDEMIOLOGICAL VARIABLES*	*EPIDEMIOLOGICAL VARIABLES*
**Age**	**Age**
≤6 months	≤6 months
7 months-14 years	7 months-14 years
15-69 years	15-69 years
≥70 years	≥70 years
**Sex**	**Sex**

*CLINICAL VARIABLES*	*CLINICAL VARIABLES*
**CSF infection previous 6 months**	**Diagnosis**
**Previous shunt**	-Traumatic brain injury
**Diagnosis**	-Vascular disease
-Traumatic brain injury	-Haemorrhagic CVA +/- intraventricular haemorrhage
-Vascular disease	-Tumour
-Haemorrhagic CVA +/- intraventricular haemorrhage	-Premature intraventricular haemorrhage
-Tumour	-Others (malformative, infectious, post-surgical causes)
-Premature intraventricular haemorrhage	**Follow-up**
-Normal-pressure hydrocephalus	
-Others (malformative, infectious, post-surgical causes)	
**Follow-up**	

*SURGICAL VARIABLES*	
**Type of surgery**	
-De novo insertion	
-Proximal revision	
-Distal revision	
-Full revision	

EVD: external ventricular drain; CSF: cerebrospinal fluid; CVA: cerebrovascular accident

### Statistical analysis

Database information was processed and analyzed by means of SPSS 15.0 for Windows.

#### Descriptive statistics

Numerical variables represented by the mean were contrasted with U Mann-Whitney test whereas those represented by the median were contrasted with Median test. Chi-square test or Exact Fisher test were used for categorical variables.

#### Analytic statistics

The proportion of infected devices as a function of the length of time they had been in place were compared between both cohorts by means of univariate Cox regression analysis and log-rank test on Kaplan-Meier estimates. Infection-free survival was analyzed by means of multivariate Cox regression model in order to estimate the simultaneous effects of independent variables on the incidence of device-related infection. Proportional hazard ratios (HRs) with 95% confidence intervals (CIs) were calculated.

A **stratified analysis **depending on the type of device placed (EVD versus shunt) was performed in order to adjust for possible confounding variables. In the multivariate analysis concerning shunts, and with the aim of preserving the test statistical power due to the low incidence of infectious events, the most relevant confounding variable was identified. Then, the independent variable to be studied (AICs) and those variables that were significant at a *p *value of 0.05 or less in the univariate Cox analysis were entered in a stepwise fashion into multivariate Cox regression models and tested for an independent effect.

All percentages were calculated per procedure. Considered level of significance was 5%. All *p *values were based on two-tailed tests of significance.

## Results

A total of 231 procedures performed in 171 patients were included for final analysis. In 112 procedures an EVD was inserted and in the remaining 119 procedures a shunt was placed, including 112 ventriculoperitoneal shunts, 2 ventriculoatrial shunts, 4 cyst-peritoneal shunts and 1 subduro-peritoneal shunt. In 119 of them AICs were employed, using non-AICs in the 112 remaining procedures. Both cohorts were homogeneous for the aforementioned independent variables except for the type of CSF shunting device employed (shunt versus EVD) and the follow-up (*Table *2). In the control cohort, EVDs percentage was significantly higher (*p *= 0.005) and, therefore, median follow-up was shorter (*p *= 0.002).

**Table 2:** Baseline characteristics of all the procedures included for analysis: Cohorts' comparison

	No. PROCEDURESS (%) (n = 231)		
**VARIABLES**	**TREATMENT COHORT (n = 119)**	**CONTROL COHORT (n = 112)**	***p *value**

**Type of device EVD**	47 (39.5)	65 (58.0)	**0.005**

**Age**:			0.600
≤*6 months*	12 (10.1)	10 (8.9)	*NS*
*7 months-14 years*	10 (8.4)	15 (13.4)	*NS*
*15-69 years*	71 (59.7)	67 (59.8)	*NS*
≥*70 years*	26 (21.8)	20 (17.9)	*NS*

**Sex Male**	68 (57.1)	64 (57.1)	1

**Diagnosis**:			0.182
*Traumatic brain injury*	8 (6.7)	10 (8.9)	*NS*
*Vascular disease*	17 (14.3)	24 (21.4)	*NS*
*CVA +/- IVH*	12 (10.1)	18 (16.1)	*NS*
*Tumour*	33 (27.7)	18 (16.1)	*NS*
*Premature IVH*	12 (10.1)	6 (5.4)	*NS*
*Normal-pressure hydrocephalus*	17 (14.3)	16 (14.3)	*NS*
*Others*	20 (16.8)	20 (17.9)	*NS*

**Follow-up, median (IQR), days**	159 (11-518)	16 (9-266)	**0.002**

n: sample size; EVD: external ventricular drain; CVA: cerebrovascular accident; IVH: intraventricular haemorrhage; IQR: interquartilic range; NS: non significant

Infection was confirmed in 29 cases, 8 of them in the treatment cohort and the remaining 21 in the control cohort (*Table *3). *Staphylococcus epidermidis *was the main causative agent (51.7%), followed by GNB (37.9%) and other agents (27.6%). Three EVDs (two of them were AICs) were infected by multiple microorganisms, being at least one of them a *Staphylococcus *spp. or a GNB.

**Table 3:** Aetiology of infections

	Treatment cohort			Control cohort			Total
		
	EVD	*Shunt*	Total	EVD	*shunt*	total	
***Staphylococcus *spp**.	**2**	**2**	**4**	**7**	**5**	**12**	16 (55.2%)

*S. epidermidis*	2	2	4	7	4	11	

*S. aureus*	0	0	0	0	1	1	

**GNBs**	**4**	**0**	**4**	**5**	**2**	**7**	11 (37.9%)

*P. aeruginosa*	0	0	0	0	1	1	

*Acinetobacter *spp.	2	0	2	1	0	1	

*Enterobacter *spp.	1	0	1	1	0	1	

*E. coli*	0	0	0	1	0	1	

*Sphingomonas *spp.	1	0	1	0	0	0	

*Klebsiella *spp.	0	0	0	2	1	3	

**Other microorganisms**	**4**	**0**	**4**	**3**	**1**	**4**	8 (27.6%)

*Enterococcus *spp.	2	0	2	2	0	2	

*Streptococcus *spp.	0	0	0	0	1	1	

*Corynebacterium *spp.	1	0	1	1	0	1	

*Candida *spp.	1	0	1	0	0	0	

**Total (infection per procedure)**	**6/47 (12.8%)**	**2/72 (2.7%)**	**8/119 (6.7%)**	**13/65 (20%)**	**8/47 (17%)**	**21/112 (18.8%)**	**29/231 (12.6%)**

EVD: external ventricular drain

**Figure 1:**
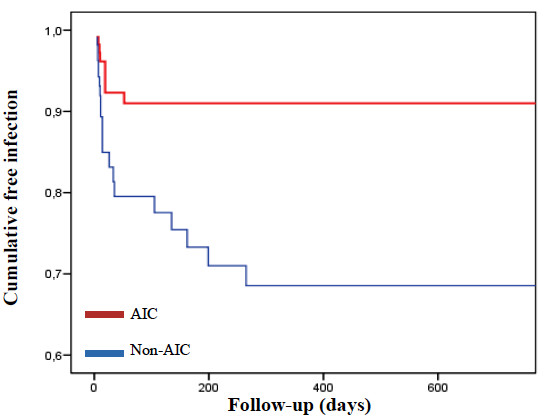
**Kaplan-Meier curve**. Cumulative freedom from infection according to antibiotic impregnation of the catheters. The risk of device-related infection was significantly lower in those procedures in which AICs were implanted compared with those procedures with non-AICs (HR 0.29; 95% CI 0.13-0.65; *p *= 0.003).

Overall infection rate was 12.6%. Univariate analysis showed significant differences between the treatment and the control cohorts (6.7% against 18.8%, respectively; *p *= 0.006). *Staphylococcal *infection rate was 6.9%, being the differences observed between both cohorts significant as well (3.4% in the treatment cohort against 10.7% in the control cohort; *p *= 0.028). However, GNB infection rate did not increase in the treatment cohort (*p *= 0.303). After adjusting for follow-up by means of Kaplan-Meier curve and univariate Cox regression analysis, significant differences were shown as well (*Figure *1). An explicative multivariate analysis using Cox regression model confirmed the decrease in overall and *Staphylococcal *infection rates when using AICs (HR 0.41; CI 95% 0.18-0.96; *p *= 0.030 and HR 0.33; CI 95% 0.10-1.05; *p *= 0.045, respectively). Thus, AICs were associated with a relative decrease of 59% in overall infection rate and a relative decrease of 67% in *Staphylococcal *infection rate. The number needed to treat (NNT) to prevent one infection was 8. The NNT to prevent one *Staphylococcal *infection was 14.

As a result of multivariate analysis an independent association between EVDs and infection risk was observed, being the likelihood of overall infection 37.03 folds higher for EVDs than shunts (CI 95% 7.25-200.01; *p *< 0.001), whereas the likelihood of *Staphylococcal *infection was 16.13 folds higher (CI 95% 2.85-90.91; *p *< 0.001) for EVDs than shunts.

All patients presented excellent tolerance to AICs. No local or systemic side-effects were described.

### Stratified analysis

A stratified analysis was performed in order to minimize the impact of the type of CSF shunting device (EVD versus shunt) on infection.

#### Shunts

**Figure 2:**
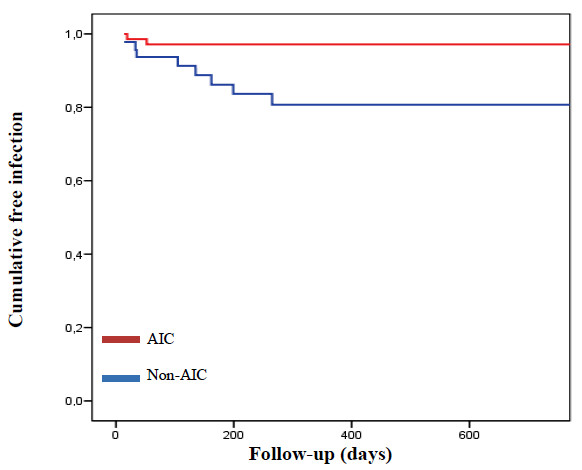
**Kaplan-Meier curve**. Cumulative freedom from infection in shunts according to antibiotic impregnation of the catheters. The risk of shunt-related infection was significantly lower in those procedures in which AICs were implanted compared with those procedures with non-AICs (HR 0.16; 95% CI 0.03-0.73; *p *= 0.018).

A total of 119 shunt procedures were included in this study, employing AICs in 72 of them (60.5%). When comparing basal characteristics of both cohorts, a significantly higher percentage of patients between 15 and 69 years old and of those who underwent de novo insertion shunts was observed in the treatment cohort, while a significantly higher percentage of patients between 7 months old and 14 years old and of those who underwent full revision of the shunt was documented in the control cohort (*Table *4). Ten infections were confirmed in this subgroup (2 in the treatment cohort and 8 in the control cohort; overall infection rate 8.4%). *Staphylococcus *spp. was the most frequent causative agent (70%), followed by GNB (20%) and other microorganisms (10%. See *Table *3). Overall infection rate was significantly lower when employing AICs (2.8% in the treatment cohort against 17% in the control cohort; *p *= 0.014). *Staphylococcal *infection rate was 5.9% (7/119). When comparing control and treatment cohorts, the rate decreased from 10.6% to 2.8% (*p *= 0.111). Only two GNB infections were confirmed (1.7%), and both of them appeared in the control cohort *(p *= 0.303). Univariate Cox regression analysis showed a relative decrease of 84% in overall infection rate (HR 0.16; CI 95% 0.03-0.73; *p *= 0.018) in AICs procedures (*Figure *2). In the multivariate analysis, the most important confounding factor (prior shunt) and the independent variable to be studied (AICs) were included in final models. Besides that, the independent variables associated with increasing infection rate after univariate analysis (prior CSF infection and full revision), were entered in a stepwise fashion. As a result, the protective effect of AICs against overall infection was significant in model 1 (HR 0.18; CI 95% 0.04-0.87; *p *= 0.016) and marginally significant in model 2 (HR 0.25; CI 95% 0.05-1.29; *p *= 0.071).

**Table 4:** Baseline characteristics of shunt procedures: Cohorts' comparison

	No. PROCEDURES (%) (n = 119)		
**VARIABLES**	**TREATMENT COHORT (n = 72)**	**CONTROL COHORT (n = 47)**	***p *value**

**Age**:			**0.027**
≤*6 months*	6 (8.3)	8 (17.0)	*NS*
*7 months-14 years*	7 (9.7)	12 (25.5)	*0.021*
*15-69 years*	40 (55.6)	16 (34.0)	*0.022*
≥*70 years*	19 (26.4)	11 (23.4)	*NS*

**Sex Male**	38 (52.7)	30 (63.8)	0.234

**CSF infection in previous 6 months**	16 (22.2)	10 (21.3)	0.903

**Previous shunt**	16 (22.2)	19 (40.4)	**0.033**

**Diagnosis**:			0.071
*Traumatic brain injury*	4 (5.6)	0 (0)	*NS*
*Vascular disease*	6 (8.3)	3 (6.4)	*NS*
*CVA +/- IVH*	1 (1.4)	0 (0)	*NS*
*Tumour*	21 (29.2)	6 (12.8)	*NS*
*Premature IVH*	6 (8.3)	5 (10.6)	*NS*
*Normal-pressure hydrocephalus*	17 (23.6)	16 (34.0)	*NS*
*Others*	17 (23.6)	17 (36.1)	*NS*

**Type of surgery**:			**0.001**
*De novo*	55 (76.4)	27 (57.4)	*0.029*
*Proximal revision*	6 (8.3)	2 (4.3)	*NS*
*Distal revision*	4 (5.5)	0 (0)	*NS*
*Full revision*	7 (9.7)	18 (38.3)	*<0.001*

**Follow-up, median (IQR), days**	384.5 (184-704.75)	385 (144-1085)	0.910

n: sample size; CSF: cerebrospinal fluid; CVA: cerebrovascular accident; IVH: intraventricular haemorrhage; IQR: interquartilic range; NS: non significant

#### External ventricular drains

**Figure 3:**
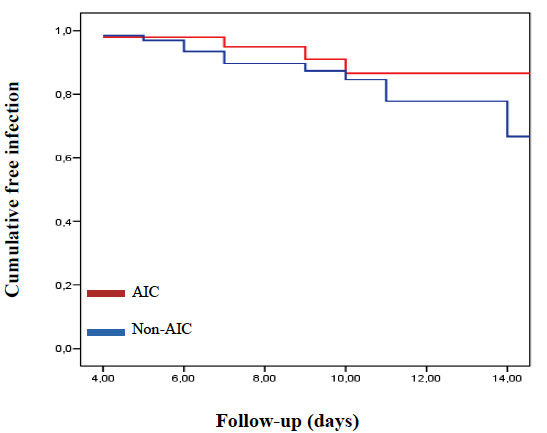
**Kaplan-Meier curve**. Cumulative freedom from infection in EVDs according to antibiotic impregnation of the catheters. The risk of EVD-related infection was lower in those procedures in which AICs were implanted compared with those procedures with non-AICs. However, the differences observed were not statistically significant (HR 0.70; 95% CI 0.26-1.87; *p *= 0.479).

A total of 112 procedures with EVDs were included, employing AICs in 47 of them (42%). Both cohorts were homogeneous when comparing basal characteristics. Median length of ventriculostomy was 9 days in the treatment cohort and 10 days in the control cohort (*p *= 0.679). Nineteen infections were confirmed in this subgroup (6 in the treatment cohort and 13 in the control cohort; overall infection rate 17%). *Staphylococcus *spp. and GNB were isolated in similar percentage of positive cultures (47.4%), while other microorganisms were isolated in 36.8% of positive cultures (*Table *3). Overall infection rate was lower in the treatment cohort (12.8%, 6/47) than in the control cohort (20%, 13/65; *p *= 0.310). *Staphylococcal *infection rate was also lower in the study cohort (4.3% against 10.8%), but these differences were not statistically significant (*p *= 0.299). Gram-negative bacilli infection rate was similar in both cohorts (*p *= 1). Kaplan-Meier curve and univariate Cox regression analysis adjusting for length of ventriculostomy did not show significant differences between both cohorts (*Figure *3).

## Discussion

Prevention strategies designed to control device-related infectious complications have been determined in the last decades by the knowledge and comprehension of the mechanisms of infection involved. Given the importance that microorganism's adherence to CSF shunting catheters surface has achieved in the pathogenesis of infection, the most promising strategies are focusing on intrinsic treatment of the catheter surface in order to enhance resistance to colonization phenomena [40].

There are a rising number of studies that support the use of rifampicin and clindamycin impregnated catheters in shunt procedures in order to reduce not only the incidence but also the costs of device-related infectious complications [11-18,25-27,29-32]. However, only two of these studies mention the effect of AICs on *Staphylococcal *infection [16,17]. Experience with EVDs is shorter, since only two studies assessing rifampicin and clindamycin impregnated catheters efficacy have been published in the literature to date [17,34]. Besides that, two more studies evaluating minocycline and rifampicin impregnated EVDs have been reported [33,35].

The results obtained in the present retrospective study show a significant reduction in infection at our centre in those cases where AICs were used (*p *= 0.006) for CSF shunting procedures. A multivariate analysis of data, confirming that AICs are helpful and safe in reducing infectious complications rate (*p *= 0.030), was achieved with the aim of minimizing the impact of possible confounding factors (the most relevant one may be the type of CSF shunting device -internal versus external-). Moreover, a significant decrease in *Staphylococcal *infection rate was observed in both univariate (*p *= 0.028) and multivariate (*p *= 0.045) analyses. The use of these catheters did not increase GNB infection rate. On the contrary, a trend to reduce the infection caused by these bacteria was observed, although the differences observed between both cohorts were not significant. In view of these results, rifampicin and clindamycin impregnated catheters in both shunt and EVD procedures are shown to be a helpful and safe tool to reduce and control the incidence of infection after their use.

Despite the fact that it was not one of the prefixed objectives of the study, multivariate analysis demonstrated that EVDs were 37.03-fold more likely to become infected than shunts, as well as 16.13-fold more likely to become infected by *Staphylococcus *spp. than shunts. Although it is widely accepted that EVDs risk of infection is higher than shunts risk, to our knowledge this is the first study that quantifies the increase in the likelihood of infection associated to the former. External ventricular drains removed before the fifth day following insertion were excluded from this study with the purpose of minimizing the false-negative rate, a bias that must be considered. However, the results hereby reported may approach the risk of infection assumed when making the decision of implanting an EVD instead of a shunt in certain patients.

A stratified analysis was performed depending on the type of CSF shunting device employed with the aim of minimizing as much as possible the impact of confounding factors. Thus, in the subgroup of shunts, rifampicin and clindamycin impregnated catheters were associated with a significant decrease in overall infection (*p *= 0.014, univariate analysis). After multivariate analysis, a marginally significant decrease in these complications was also observed, with a relative reduction of 75% in overall infection rate when employing AICs. These differences have also been reported by other authors [16,29]. *Staphylococcal *infection rate was importantly reduced too -from 10.6% to 2.8%- when inserting AICs. However this result was not statistically significant. This fact could be explained by the low incidence of infectious events, being necessary to increase the sample size to obtain statistically relevant differences. Besides that, the use of AICs did not increase GNB infection, a similarity observed in other studies results [17,25].

Considering exclusively EVDs, AICs were associated with a decrease from 20% to 12.8% in overall infection rate and a decrease from 10.8% to 4.3% in *Staphylococcal *infection rate (*p *= 0.310 and *p *= 0.299, respectively), without increasing GNB infection rate. Once observed the results obtained after multivariate analysis of the whole sample, these findings could be explained by the small sample size given the low incidence of infectious events.

There are only two studies that have been published in the literature to date which compare the incidence of infection when employing rifampicin and clindamycin impregnated EVDs against control catheters [17,34]. Despite the fact that both studies point to the usefulness of these devices in reducing infectious complications, they include a small number of procedures. Besides that, the study carried out by Tamburrini et al. [34] includes a specific subgroup of patients suffering from hydrocephalus secondary to posterior fossa tumours. Similarly, the reports with minocycline and rifampicin impregnated catheters show a significant reduction in infection associated with the use of treated catheters [33,35]. However, a recent study reports a significant number of false-negative results from minocycline and rifampicin impregnated EVDs compared with non-antibiotic impregnated EVDs [41]. This potential bias has not been studied concerning rifampicin and clindamycin impregnated catheters.

Another consideration is that the presence of infection involves not only morbidity and mortality for the patient, but increasing costs for the hospital as well. One of the latest lines of research that remains incomplete is to determine if AICs reduce infection hospital costs, despite their higher price when comparing to non-AICs. Economical differences will be probably important, given the increased cost derived from treating a CSF device-related infection when considering intravenous antibiotic treatment, intensive care unit stay, new CSF shunting device or prolonged hospital stay. Although the difference in price between AICs and non-AICs is easily measurable (differences around 100 € in EVDs and 180 € in shunts), it is not so easy to quantify the hospital cost derived from an infectious event. This difficulty may be the main obstacle in designing a study to assess these costs. Three studies have been published in the literature in order to assess the impact of antibiotic-impregnated shunts on hospital costs. All of them suggest the economic effectiveness of the aforementioned systems [13,14,30]. However, no study has been performed with EVDs with this purpose, thus the economical impact of AICs in this field remains unknown.

Finally, the main limitations of this study derive from its retrospective design, as it does not provide the best level of scientific evidence to draw conclusions. Multivariate and stratified analyses were performed in order to avoid the impact of possible confounding factors. Besides that, data were exclusively collected by the first author of this study with the aim of giving consistency to data codification and in order to make database management easier.

## Conclusions

The results obtained in this retrospective study suggest that AICs are a protective and safe tool against infection and, specifically, against *Staphylococcus *spp. infection. The number needed to treat for AICs is 8 to prevent one infection and 14 to prevent one staphylococcal infection. When comparing with shunts, the use of EVDs is associated with a 37-fold increased likelihood of infection. However, further prospective, randomized, controlled trials are required to confirm these results.

## Competing interests

The authors declare that they have no competing interests.

## Authors' contributions

RGG carried out data collection, participated in the design of the study and drafted the manuscript. GRB: conceived of the study, and participated in its design and coordination and helped to draft the manuscript. CFP: participated in the design of the study and performed the statistical analysis. NdP: participated in the design of the study and performed the statistical analysis. All authors read and approved the final manuscript.

## Pre-publication history

The pre-publication history for this paper can be accessed here:

http://www.biomedcentral.com/1471-2377/10/93/prepub
